# A Hybrid Smartphone Indoor Positioning Solution for Mobile LBS

**DOI:** 10.3390/s121217208

**Published:** 2012-12-12

**Authors:** Jingbin Liu, Ruizhi Chen, Ling Pei, Robert Guinness, Heidi Kuusniemi

**Affiliations:** Department of Navigation and Positioning, Finnish Geodetic Institute, Geodeetinrinne 2, Masala 02431, Finland; E-Mails: ruizhi.chen@fgi.fi (R.C.); ling.pei@fgi.fi (L.P.); robert.guinness@fgi.fi (R.G.); heidi.kuusniemi@fgi.fi (H.K.)

**Keywords:** smartphone positioning, mobile LBS, probabilistic algorithms, sensor fusion, ubiquitous computing

## Abstract

Smartphone positioning is an enabling technology used to create new business in the navigation and mobile location-based services (LBS) industries. This paper presents a smartphone indoor positioning engine named HIPE that can be easily integrated with mobile LBS. HIPE is a hybrid solution that fuses measurements of smartphone sensors with wireless signals. The smartphone sensors are used to measure the user’s motion dynamics information (MDI), which represent the spatial correlation of various locations. Two algorithms based on hidden Markov model (HMM) problems, the grid-based filter and the Viterbi algorithm, are used in this paper as the central processor for data fusion to resolve the position estimates, and these algorithms are applicable for different applications, e.g., real-time navigation and location tracking, respectively. HIPE is more widely applicable for various motion scenarios than solutions proposed in previous studies because it uses no deterministic motion models, which have been commonly used in previous works. The experimental results showed that HIPE can provide adequate positioning accuracy and robustness for different scenarios of MDI combinations. HIPE is a cost-efficient solution, and it can work flexibly with different smartphone platforms, which may have different types of sensors available for the measurement of MDI data. The reliability of the positioning solution was found to increase with increasing precision of the MDI data.

## 1. Introduction

Smartphone indoor positioning technology is a boost to the rapidly growing mobile location-based services (LBS) industry. As the latest initiative, the In-Location Alliance, formed by 22 member companies, including Nokia, Qualcomm, Broadcom, *etc.*[1], was recently launched to drive innovation and market adoption of high-accuracy indoor positioning and related services. The continued development of accurate and reliable LBS will not only improve the experience of smartphone users, but will also create new marketing opportunities. Emerging indoor LBS include social networking, people finders, marketing campaigns, asset tracking, *etc.* Because most indoor LBS are used by pedestrians, in this work we focus the development of our proposed indoor positioning solution on a pedestrian scenario.

Multiple sensors and signals of opportunity have been used for indoor positioning and navigation [2,3]. Examples of such sensors include accelerometers, gyroscopes, compasses, cameras, proximity sensors, and electromyography sensors [4]. In this work, signals of opportunity are defined as signals that were not originally intended for positioning and navigation, and they include radio frequency (RF) signals, e.g., cellular networks, wireless local area networks (WLAN) and Bluetooth [5], and naturally occurring signals, such as Earth’s magnetic field and polarized light from the sun [6]. Each method has its own respective drawback. For example, cellular positioning systems offer limited accuracy. Inertial sensors only provide a relative location estimate with accuracy degrading over time, and they must be used together with other technologies, e.g., Global Positioning System (GPS), to estimate absolute location [7,8].

Due to the cost effectiveness and extensive availability of the existing network infrastructure, WLAN signals have been widely used for indoor positioning [9–11]. Traditional solutions usually have utilized a special-purpose hardware unit to observe the WLAN received signal strength indication (RSSI) signals for indoor positioning. RSSI observables are location dependent, and they are commonly used to estimate indoor locations through a fingerprinting approach.

This study develops a smartphone indoor positioning solution using the built-in hardware and computational resources of smartphones. Significant advantages of re-using an existing smartphone platform for positioning include cost efficiency of the positioning solution and the effective combination of measurements from multiple sensors and signals for enhanced positioning performance. Further, the smartphone-based positioning solution is more convenient for integration with related applications and services because smartphones have become a common platform for mobile LBS.

A major challenge in the fingerprinting approach is the large variance of RSSI observables caused by the significantly non-stationary nature of WLAN signals. Most of the previous WLAN positioning solutions pursued the position estimation problem as single-point positioning in which positions were considered as a series of isolated points [11–13]. In the single-point positioning approach, the results are vulnerable to RSSI variance, and the positioning accuracy and reliability are degraded significantly. To mitigate the impact of RSSI variances, the position estimate can be augmented by motion information because the dynamics of indoor users are usually restricted, and their locations are highly correlated over time. Location changes over time are represented in this paper as motion dynamics information (MDI) such as the distance moved and movement direction and/or direction change. In our approach, MDI is physically measured using the smartphone sensors, and MDI is further integrated with RSSI observables through the methodology of hidden Markov models (HMM). RSSI measurements and the corresponding media access control (MAC) addresses can be obtained without an authenticated link. Thus, WLAN positioning can be performed autonomously, avoiding the privacy concerns that typically arise in other positioning techniques. Further, the positioning functionality can be operated in conjunction with communication services, which facilitates the deployment of related applications and services.

In contrast to previous studies, which commonly utilized simplified motion models, e.g., a linear model, to represent a user’s motion [13–17], our approach uses smartphone sensors to measure the real motion of a user. Because the motion of an indoor user is usually quite complicated and he/she can change motion states at any time, e.g., stationary, walking, walking speed change, direction change, and even sudden turnaround, existing models are not capable of describing user motion accurately. By taking advantage of multiple sensors in a smartphone, our proposed solution measures MDI more accurately, and our solution is more effective for situations in which different motion states occur.

The utilization of the HMM methodology incorporates motion dynamics information into RSSI positioning, and it allows for the use of current RSSI measurements in the position estimate as well as historical information regarding the position estimate. For two reasons, the HMM is preferred for the integration of different types of measurements in this study. First, Markov processes do not impose any deterministic form of models to restrict the user’s movement, and they are hence widely suitable for the representation of complicated motion processes of indoor users. Second, the methodology of HMM has been well-developed in mathematics, and the solutions of HMM problems can be used effectively in a smartphone platform to resolve position estimates.

The proposed solution is a hybrid data fusion solution: the WLAN RSSI observables are fused with the measured MDI. The hybrid fusion approach, named HIPE (hybrid indoor positioning engine), was developed with a Nokia N8 smartphone and is currently being implemented with other smartphone platforms. Because different smartphone platforms may have different combinations of sensors available for the measurement of MDI, this paper presents methods for dealing with different scenarios in which different types of MDI are available.

In summary, this paper proposes a smartphone indoor positioning engine that can be easily integrated with mobile LBS. Our positioning solution is a data fusion scheme, and it uses the smartphone built-in sensors to physically measure the motion dynamics information of indoor users. In comparison with previous works, our solution is more widely applicable for various motion scenarios and different smartphone platforms. Two algorithms of HMM problems, *i.e.*, the grid-based filter and the Viterbi algorithm, are applied in this paper to resolve position estimates.

The rest of this paper is organized as follows: Section 2 provides an overview of the related research in WLAN positioning and pedestrian motion estimation using smartphone sensors; Section 3 presents the methods of measuring pedestrian MDI with a smartphone. The proposed positioning solution is presented in Section 4. Section 5 evaluates the proposed solution with experimental results. Finally, Section 6 concludes the paper and provides directions for future work.

## 2. Background of the RSSI-Based WLAN Positioning and Pedestrian Motion Dynamics Estimate

Two basic approaches are used for the estimation of locations with WLAN RSSI measurements. The trilateration-based approach first translates RSSI measurements into the distances between a mobile user and multiple access points (APs) based on a radio propagation model and then calculates the user’s location using the obtained distances and AP coordinates [18]. The major challenges in this approach include the large errors associated with estimated distances and difficulties in system deployment, e.g., the trouble associated with obtaining the AP coordinates indoors. In contrast, the fingerprinting approach determines a user’s position by matching RSSI measurements with a fingerprint database in a deterministic or stochastic way. The *k*-nearest neighbors (KNN) method employs a deterministic approach to estimate a location [19,20], which is the centroid of the *k* closest neighbors, in terms of the Euclidean distance between the online RSSI measurements and the RSSI measurements in the database. The stochastic methods impose a probabilistic model on the online RSSI measurements and calculate the posterior probability distribution [11–13]. Different probabilistic models have been used in previous studies, ranging from a simple Gaussian model to more complex kernel functions [21–23].

Many of the previous methods used memory-less or single-point positioning approaches, which only utilize current RSSI measurements discretely for determining a position estimate [10–12]. The accuracy and reliability of single-point positioning solutions are degraded by the non-stationary nature of RSSI, due to the multipath and non-line-of-sight propagation of WLAN signals. The studies presented in [13,24–28] show that positioning accuracy can be improved by the incorporation of current RSSI measurements in conjunction with knowledge of motion dynamics and historical measurements.

Motion dynamics describe the correlation of the spatial coordinates of user positions over time. In previous studies, two approaches have been proposed to use motion dynamics information for improving positioning accuracy. One approach represents a user’s motion with a set of predefined motion models, which describes the time evolution of the user’s positions [13–17]. The other approach uses a map to restrict the potential direction of motion and the space of the user [14,15]. Based on both approaches, a form of Bayesian filters has been used to perform position estimation. For example, Kushki *et al.*[13] used a linear motion model to describe the motion of an indoor user and utilized a nonparametric information (NI) filter to resolve position estimates. An alpha-beta (αβ) filter was also used for positioning based on a constant speed motion model [29,30]. Au *et al.*[14] assumed a linear motion model and performed a map-adaptive Kalman filter (KF) to estimate positions, while the position accuracy was improved by resetting the KF when the user is located at an intersection on a map. Particle filters can further improve positioning accuracy by applying more sophisticated non-linear and non-Gaussian models, as well as map information [15,31–36].

The applicability of these solutions is restricted by the fidelity of the motion models. When considering general users, for example, in an office or in a shopping center, the commonly used models are insufficient for the representation of indoor user motion, which may involve abrupt turns or stops. The motion dynamics of a pedestrian user are especially complex: user motion is governed by decision models, purpose of the movement, choice of destination, and interactions with other people or objects in the environment. An incorrect model results in inaccurate estimates [14]. Map data can only provide static information, e.g., potential movement directions and intersections and are incapable of presenting real-time motion status. For example, a pedestrian may turn around suddenly in a corridor. Furthermore, in the specific cases given in previous studies, the utilization of map data was based on a unique layout of the indoor environment. As a result, these solutions must process the map data of different indoor environments on a per-case basis, and they cannot be applied universally until a unified method for obtaining indoor map information exists. To make our solution widely applicable, this paper does not include the utilization of map data in the proposed solution, although map data also can be used to improve the positioning accuracy further.

In our smartphone positioning solution, the built-in smartphone sensors offer the ability to physically measure the motion dynamics information, including the distance moved and the heading. Three types of methods have been developed in past works to measure pedestrian distance. One is direct foot-to-foot step length estimation using a six-degree-of-freedom inertial measurement unit (IMU), installed on the feet [8,37]. The second method uses radio sensors, such as an ultrasound sensor, usually installed on the feet as well, to directly measure foot-to-foot ranges [38]. The third method monitors the occurrence of step events and estimates step lengths based on the periodic acceleration pattern of a pedestrian user. Pedestrian acceleration can be measured using accelerometers, and the features of the acceleration pattern, such as the magnitude of the total acceleration and its periodic pattern, are closely correlated with the pedestrian dynamics, e.g., the motion states and the walking speed. The third is preferable for smartphone positioning, while the others are usually used for special applications in dedicated positioning systems.

Heading can be determined using two approaches. An absolute direction can be directly measured or estimated by sensors, such as a digital compass or GPS, while a relative change in heading can be measured by gyroscope sensors. A relative heading change can be further used to calculate an absolute heading based on a previously determined heading. The first approach is attractive because it directly produces an absolute heading. However, GPS depends on the visibility of signals in space and is usually not available indoors. A digital compass is self-contained, and it can output measurements ubiquitously. Digital compasses, however, are susceptible to errors, including effects from electric devices and steel structures, and calibration and filtering processing are needed to improve compass accuracy.

On the contrary, a gyroscope can be used to measure a relative direction change with no impacts from the environment. As the gyroscope measurements are integrated over time, however, the error increases over time; hence, an external reference is needed for periodic calibration. To further improve heading results, motion recognition methods can be used to detect motions that may cause heading changes. For example, a U-turn may indicate a rotation of 180° [39,40].

This paper employs a 3-axis accelerometer and a digital compass, which are available in the smartphone platform used (Nokia N8), to measure MDI data at run time. We limit the scope of this work to MDI estimation using these two sensors to demonstrate the effectiveness of the proposed positioning solution. Other sensors and techniques of MDI estimation, e.g., vision-based techniques, will be integrated with HIPE in the future.

## 3. Sensing Motion Dynamics with a Smartphone

Motion dynamics are defined in this paper as position changes over time, which are represented by the distance moved and the movement heading. Smartphone sensors can be used in different capacities to measure the motion dynamics of a user. Traditionally, one method to estimate changes in location and direction is by using an IMU, which typically consists of accelerometers, gyroscopes and/or compasses. In a platform where the attitude is known, acceleration measurements are integrated once to determine speed and twice to determine travelled distance. The movement heading of the platform is observed by a gyroscope and a compass, which provide the measurements of heading change and absolute heading, respectively. However, this approach is not applicable to the scenario of smartphone users. Built-in smartphone sensors are commonly low-cost and have worse performance than traditional IMUs. Furthermore, the integration operation is not suitable for smartphone pedestrian scenarios due to the lack of knowledge about the platform’s attitude. It is not practical for a pedestrian user to maintain the device in a fixed attitude, and it is also complicated to estimate a changing smartphone attitude using the built-in sensors.

**Figure 1.**
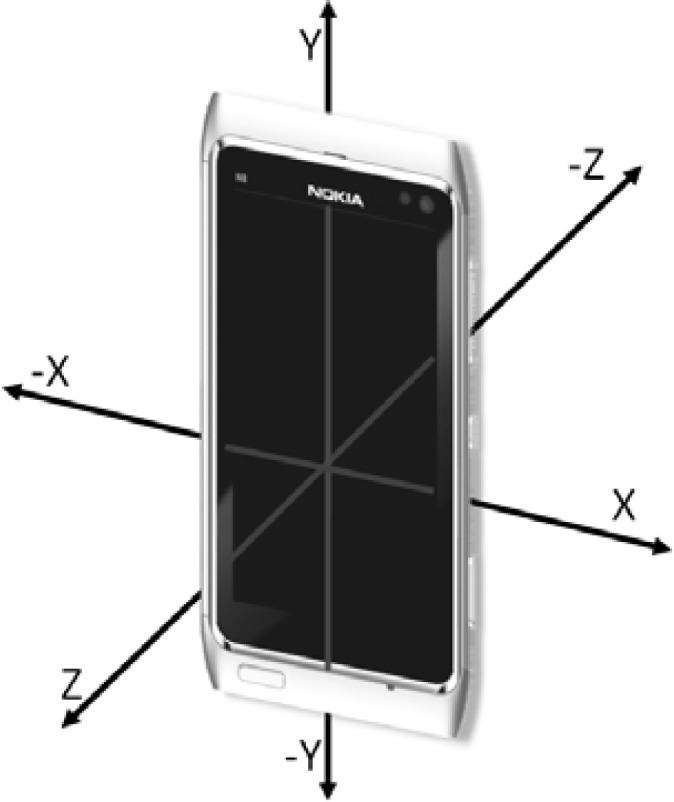
The smartphone body frame defined for the Nokia N8 consists of three axes and six directions, and it uses the right-hand Cartesian coordinate system. The various sensors all use the common body frame.

Various sensors included in modern smartphones can provide multiple approaches to measure the MDI of a pedestrian user. This section presents the methods used in this study to estimate the distance moved and heading using an accelerometer and a digital compass, respectively. Presentation of how the estimated MDI is used in the proposed positioning solution follows in the section after. Figure 1 shows the three axes and six directions of the device body frame of the smartphone platform. The body frame uses the right-hand Cartesian coordinate system [41].

### 3.1. Moved Distance

**Figure 2.**
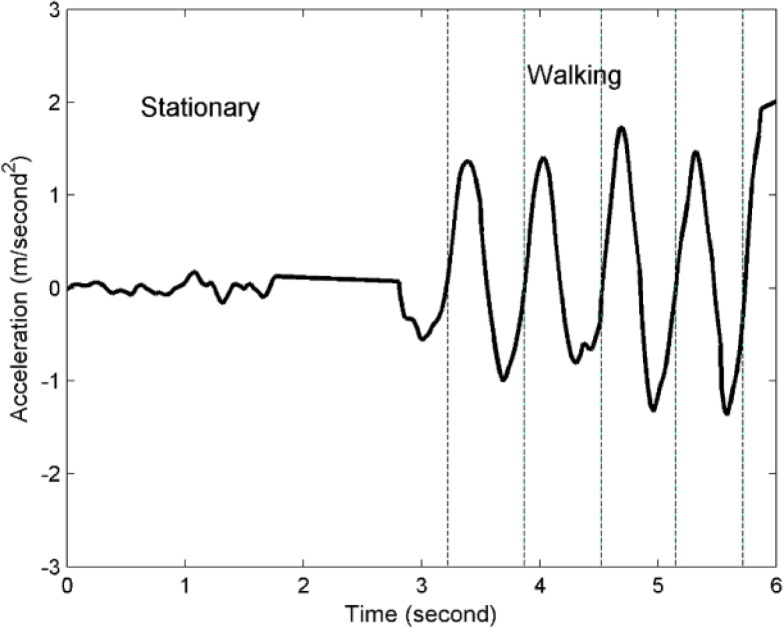
The acceleration patterns of a pedestrian in stationary and walking states.

In this study, the pedestrian motion distance is estimated using two procedures: step detection and step length estimation, both of which are widely used in the pedestrian dead reckoning (PDR) approach. The fundamental idea behind the PDR approach is derived from pedestrian acceleration characteristics [42]. Figure 2 shows typical acceleration patterns in stationary and walking states.

A walking step event can be explicitly divided into two phases. In the first phase, one foot of a pedestrian is in contact with the ground, and in the shorter second phase, both feet are in contact with the ground. Step detection is used to identify these two-phase step events. Once the step events have been detected, the step length and the step heading of particular steps are determined subsequently.

The pedestrian acceleration characteristics are measured with a smartphone three-axis accelerometer, which outputs a three-dimensional (3D) composite acceleration vector due to Earth’s gravity and pedestrian acceleration. It is difficult to separate pedestrian acceleration from that of gravity because the sensor attitude is unknown. In the PDR approach, the norm of 3D acceleration is used to detect the step events and estimate the step length. When the device is stationary, the magnitude of the gravity is learned by taking the average of the acceleration norm for a certain period, e.g., 1 s. Then, gravity is separated from the acceleration norm to obtain the pedestrian acceleration as follows:

(1) $${\left\|a_{p}\right\|}_{t}={\left\|a\right\|}_{t}-\hat{g}$$

where ĝ is the measured value of the Earth’s gravity, and ||*a_p_*||*_t_* is the pedestrian acceleration.

Using the acceleration measurements, step detection and step length estimation can be accomplished through different methods [43,44]. In this study, the Nokia N8 smartphone outputs accelerometer measurements at a rate of 35 Hz. The values of pedestrian acceleration are first calculated by Equation (1). Then, they are processed with a sliding window for smoothing, and the window length is nine measurements (equal to roughly 0.25 s). The smoothed results are used for motion state recognition and step detection through peak detection and zero-crossing algorithms, which can be found in [43,44]. When step events are detected, the length of each step is estimated using a constant model [45]. The constant model uses an empirically-derived constant value of the step length (70 cm per step in this paper), based on generic pedestrian motion.

### 3.2. Heading

The heading is measured directly in this study with the digital compass of the smartphone. According to the phone’s software development kit (SDK) documents [46], the built-in compass outputs the azimuth of the device as degrees from magnetic north in a clockwise direction with respect to the Y-axis shown in Figure 1.

Compasses are susceptible to magnetic interferences and must be calibrated after being placed near anything that bears a magnetic force. The accuracy of a compass may be affected by any nearby ferromagnetic materials. In the SDK [46], the status of calibration is indicated by a number from 0 to 1. A value of 1 is the highest level that the device can support, and 0 is the worst. If the device is not calibrated, the azimuth may be inaccurate. The device is calibrated by rotating it through all of its axes, e.g., rotating the device in a number eight pattern [47].

This paper evaluates compass measurements of the smartphone in real indoor dynamic environments, and the obtained values of accuracy are used as tolerance thresholds in the proposed positioning solution.

Using the distance moved and the heading, user locations are correlated over time. Current positions are recursively propagated in a locally horizontal frame during a successive process from a previously determined position as follows:

(2) $${\left[\begin{array}{l} E\\ N \end{array}\right]}_{t}={\left[\begin{array}{l} E\\ N \end{array}\right]}_{t-1}+\left[\begin{array}{l} d_{t}\;\text{sin}(\alpha_{t})\\ d_{t}\;\text{cos}(\alpha_{t}) \end{array}\right]$$

where *t* is the epoch time, *E*, *N* are the east and north coordinate components in the locally horizontal (East-North-Up, ENU) frame, respectively. *d_t_* and *α_t_* are the distance moved and the heading during the current epoch.

## 4. Hybrid Indoor Positioning Solution in the Smartphone

**Figure 3.**
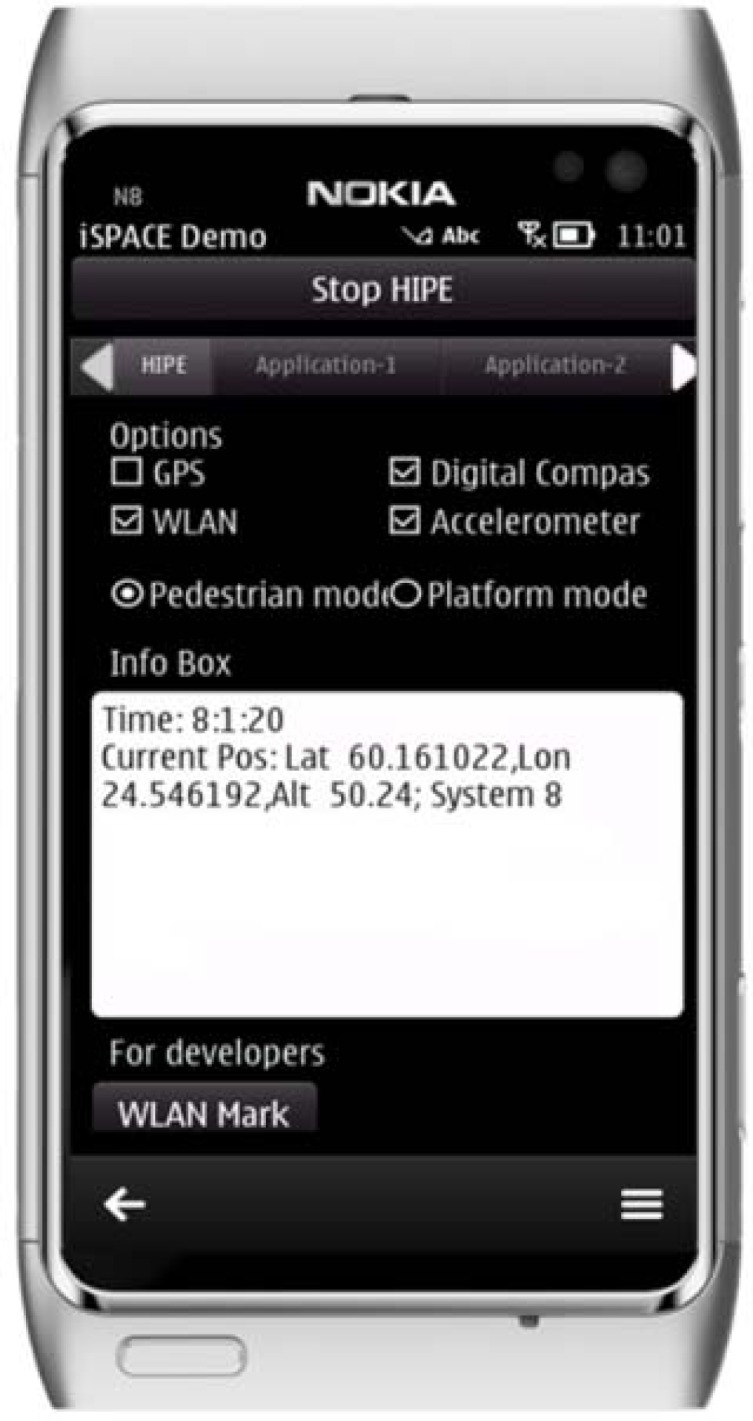
The interface of the HIPE allows developers to select the sensor options. The graphical interface is not required when the engine is embedded into a specific application.

This section presents the proposed smartphone positioning solution, named hybrid indoor positioning engine (HIPE). The proposed HIPE solution was implemented with a Nokia N8 smartphone. The device runs on the Symbian^3 operating system (OS), and it has a CPU (central processing unit) clock rate of 680 MHz (ARM™ 11) and an internal memory of 135 MB [48]. The built-in WLAN, accelerometer and compass are used in this study. The Qt SDK and Qt Creator IDE (integrated development environment) are used for software development [41,46,47]. Figure 3 shows the graphical interface of the HIPE.

**Figure 4.**
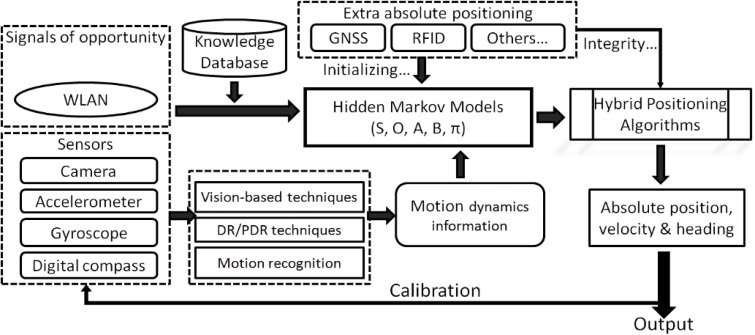
The general high-level architecture of the HMM solution that fuses the measurements of the sensors and WLAN to estimate the absolute positions.

The methodology of HMM is adopted in this study to fuse the MDI data, the current RSSI observables and the historical information of position estimates. Figure 4 shows the general architecture of the proposed HIPE solution. This section also presents the flexibility of the HIPE, which can work with different combinations of MDI.

This section first introduces the fundamentals of hidden Markov models and the related solutions of HMM problems, and it then presents the methods of position estimation based on HMM with an emphasis on the utilization of MDI to augment WLAN positioning.

### 4.1. Hidden Markov Models and the Solutions

The concept of hidden Markov models arises from the well-known Markov model in which each state corresponds to a physically observable symbol. Observable Markov models are too restrictive for application to many problems of interest because they require each state to be directly observed. Subsequently, the concept of Markov models is extended to include the case of hidden Markov models, in which states are not directly observable (hidden), and an observation is a probabilistic function of the hidden states. In the HMM, the underlying stochastic process (state evolution) is not directly observable, but it can be observed in the Bayesian sense through another set of stochastic processes, which produce the sequence of observables. Hidden Markov models are significantly more applicable in the real world than observable Markov models when physical states of interest are largely unobservable. The basic theory and selected applications of HMM have been presented with details in [45,49]. For the sake of completeness, this section introduces the related fundamentals briefly.

**Figure 5.**
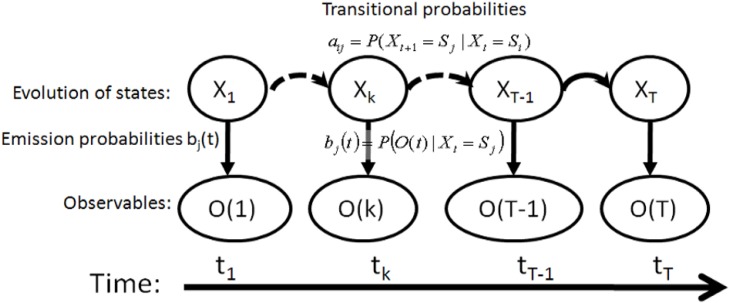
The representation of a physical system by a hidden Markov model.

A general hidden Markov model characterizes a physical system with a state-space model, as shown in Figure 5. Formally, an HMM includes five elements, given as follows [49]:

1. *S*, the state space that consists of *N* hidden states *S* = {*S*_1_, *S*_2_, …, *S_N_*}.
2. *O*, a set of observables at epoch *t O*(*t*) = {*o*^1^, *o*^2^, …, *o^M^*}, where *M* is the number of observable symbols.
3. *A*, the matrix of state transition probabilities *A* = {*a_ij_*}. A state transition probability *a_ij_* defines the probability that the state transits from a value *S_i_* at the immediately prior epoch to another value *S_j_* at the current epoch, *i.e.*, *a_ij_= P*(*X*_*t*+1_ = *S_j_|X_t_* = *S_i_*), 1 ≤ *i*, *j* ≤ *N*.
4. *B*, the matrix of emission probabilities, *B* = {*b_j_*(*t*)}, where *b_j_*(*t*) = *P*(*O*(*t*)|*X*(*t*) = *S_j_*) 1 ≤ *j* ≤ *N*.
5. *π*, an initial state probability distribution *π* = {*π_i_*}, where *π_i_* defines the probability that the state has a value *S_i_* at the first epoch, *i.e.*, *π_i_* = *P*(*X_1_* = *S_i_*) 1 ≤ *i* ≤ *N*.

The principle of HMM has been used in numerous applications, and the evaluation problems associated with HMM can be categorized into three groups: the estimation of the probability (or likelihood) of an observable sequence given a specific HMM; the determination of a best sequence of model states, given an HMM and an observation sequence; and the learning of model parameters to best account for the observed signals [45,49].

In the problem of position estimation, a hidden Markov model represents the temporal correlation of a user’s positions without the restriction of any particular forms of motion models. The solution of the position estimate acts as the central processor for data fusion to combine MDI data and RSSI observables. In this paper, two algorithms of HMM problems, *i.e.*, the grid-based filter and the Viterbi algorithm, are proposed to resolve position estimates for different types of applications. The details of both solutions can be found at pages 173–175 in [45].

The grid-based filter solution gives the state estimate that has the maximum posterior probability, while the Viterbi algorithm produces the most likely state sequence that has produced the observable sequence [50]. The two algorithms have distinct interpretations from each other, although they both produce the position estimate. Given the hidden state space has a finite number of states, e.g., reference points in the positioning problem, the grid-based filter algorithm produces an optimal estimation of each current epoch using historical information and current observations [51], whereas it does not necessarily produce the most likely state sequence for all epochs. In other words, the difference in the position estimates obtained by the two algorithms is described as follows: the Viterbi algorithm recalculates the entire sequence when every new observation (evidence) is obtained, while the grid-based filter algorithm directly appends a current optimal state estimate to the previously generated sequence. For location-based applications, one of the two algorithms is selected according to the situation of a specific application. For example, real-time navigation requires the grid-based filter to estimate an optimal state for up-to-date time instants, while an application of location tracking may prefer the Viterbi algorithm to produce the most likely position trajectory over the whole time period.

### 4.2. Augmenting WLAN Positioning with MDI

In the HMM approach, a user’s positions are the hidden states to be estimated, and the sequence of positions has the Markov property. Observables in this study are WLAN RSSI, and the emission probabilities of observables are probabilistic RSSI-position dependent, obtained from a knowledge database, which is created in a prior learning phase and uses a parameterized Weibull function to represent the RSSI-position dependence [21,52,53]. In the HMM approach, the calculation of state transition probabilities is a critical issue to improve the positioning performance. The positioning solution increases in reliability with precision of the state transition probabilities.

#### 4.2.1. Utilizing MDI to Calculate the State Transition Probabilities

In the position estimation problem, the state transition probability of a pair of states, *i.e.*, the reference points (RPs), is determined based on the coherence level between the user’s real motion trajectory and the relative location of the concerned two RPs. In principle, the state transition probabilities *a_ij_* meet the following properties:

(3) $$a_{\mathrm{ij}}\geq 0$$

(4) $$\sum_{j=1}^{N}a_{\mathrm{ij}}=1$$

In our HIPE solution, the state transition probabilities are refined by MDI data. The shortest accessible distance and direction between any two states are calculated with their coordinates and are stored as a look-up table in the prior knowledge database. The utilization of a look-up table reduces the computational complexity of the online positioning phase. It should be noted that a physically accessible route is usually bounded by the layout of an indoor environment. For example, one cannot walk through a wall.

A user’s MDI, including the distance moved and the heading, is measured at run-time by the smartphone accelerometers and compass, respectively, and it is then compared with the distance and direction for a pair of state candidates related to the previous and current epochs, which are looked up from the knowledge database. As a result, a higher state transition probability (*a^h^*) is determined for the state pairs that have a distance and direction consistent with the measured values, and the other state pairs are associated with the lower state transition probability (*a^l^*). In the proposed solution, the values of high and low transition probabilities are calculated as follows:

(5) $$a^{h}=K\cdot a^{l}$$

(6) $$\sum_{j=1}^{N}a_{\mathrm{ij}}=I\cdot a^{h}+(N-1)\cdot a^{l}=[N+(K-1)\cdot I]\cdot a^{l}=1$$

where *K* is the ratio between the high and low values of transitional probabilities, and *I* is the number of state pairs that have higher transitional probabilities.

In the HIPE solution, the value of *K* is adaptive to the reliability of the MDI and positioning solution of the previous epoch. The *K* value is adjusted every epoch, and it is evaluated greater when the previous positioning solution and the current MDI are more reliable, and *vice versa*.

#### 4.2.2. Flexibility for Different Situations of MDI Availability

To make the HIPE positioning engine usable with different smartphone platforms, it is necessary to address different situations of MDI availability because different smartphone platforms may have different sensors available. This subsection presents the flexibility of the HIPE solution to cope with situations when either partial or no measured MDI data are available.

Table 1 gives four different scenarios, each with a different level of MDI data available. When an accelerometer can be used, the distance moved is estimated based on the accumulated step lengths. Otherwise, based on limited indoor dynamics, the moved distance range of an indoor user can be estimated with an empirical maximum speed model, e.g., 1 m/s in this work for the scenarios “Measured heading & assumed speed” and “Assumed speed”. When a compass can be used, the heading is measured directly, otherwise the heading remains unknown, e.g., in the scenarios “Measured distance” and “Assumed speed”, and all directions are considered as possible headings because the user may change his/her heading at any time.

**Figure 6.**
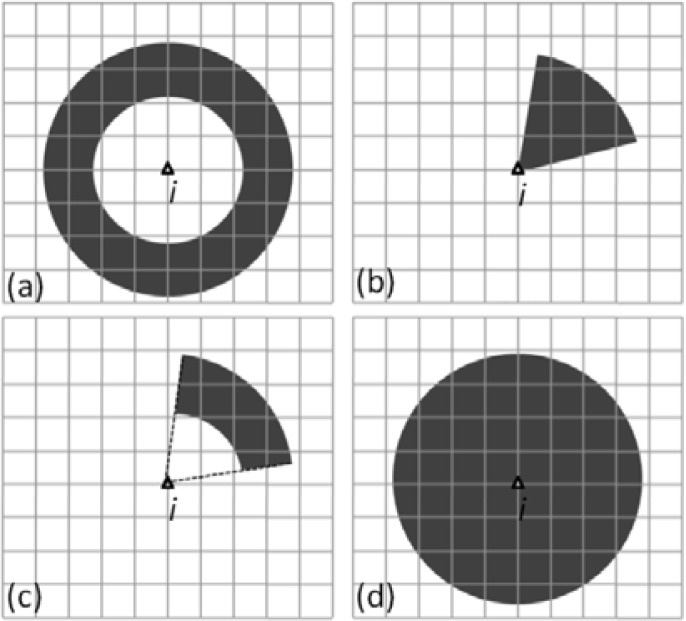
The grid points in the black areas indicate candidate states that have higher transitional probabilities based on the different combinations of MDI available. The other grid points indicate candidate states of lower transitional probabilities. The triangle point *i* is the assumed state of the previous epoch. The sub-plots (**a–d**) illustrate respectively the four scenarios given in Table 1: (**a**) denotes the scenario that uses measured distance, (**b**) denotes the scenario that uses measured heading and assumed maximum speed, (**c**) denotes the scenario that use measured distance and heading, (**d**) denotes the scenario that uses an assumed maximum speed.

The four scenarios given in Table 1 cover all of the possible situations regardless of the sensor types used. The calculation of state transition probabilities is shown in Figure 6 for the different MDI availabilities. The grid points in Figure 6 denote all possible state candidates for the current epoch, and the point *i* (the triangle) is a state candidate of the previous epoch.

When only the distance moved is measured (the scenario “Measured distance” in Table 1), the subset of state candidates (reference points) *C*_Dist_ defined by Equation (7) has higher transitional probabilities, while the others have lower transitional probabilities:

(7) $${\text{C}}_{\text{Dist}}=\{j|d-ɛ<\left\|P_{j}-P_{i}\right\|<d+ɛ,j\in 1,\cdots ,N\}$$

where *P_j_* is the coordinate of state candidate *j*, *P_i_* is the coordinate of state candidate *i*, *d* and *ε* are the measured movement distance and its uncertainty, and ||·|| is the distance between two RPs.

For this case, the subset *C*_Dist_ of state candidates is located within the ring zone around the point *i*, as shown in Figure 6(a). The radius and width of the ring are determined based on the measured distance *d* and its uncertainty *ε*, respectively.

When only the heading is measured and an empirical constant speed model is used to calculate a maximum walking range within a time interval (the scenario “Measured heading & assumed speed” in Table 1), the subset of state candidates *C*_Heading_ defined by (8) have higher transitional probabilities, while the others have lower transitional probabilities:

(8) $${\text{C}}_{\text{Heading}}=\{j|\left|{∠}_{i}^{j}-\alpha \right|<\gamma \cap \left\|P_{j}-P_{i}\right\|<\rho ,j\in 1,\cdots ,N\}$$

where *α* and *γ* are the measured heading and its uncertainty, ${∠}_{i}^{j}$ is the true direction between the *i-th* and *j-th* RPs, and *ρ* is the calculated maximum walking range within the epoch time interval.

For this case, the subset *C*_Heading_ of the state candidates is located within the sector zone radiating from point *i*, as shown in Figure 6(b). The angle of the sector zone is determined based on the uncertainty *γ*.

When both the distance moved and the heading are measured (the scenario “Measured distance & heading” in Table 1), the intersection of *C*_Dist_ and *C*_Heading_ is the subset of state candidates possessing higher transitional probabilities, as defined by Equation (9):

(9) $${\text{C}}_{\text{Dist}\&\text{Heading}}=\left\{j|j\in \left({\text{C}}_{\text{Dist}}\cap {\text{C}}_{\text{Heading}}\right)\right\}$$

In this case, the subset *C*_Heading_ of state candidates is located within the intersection zone of the ring and the sector area, as shown Figure 6(c).

Finally, when neither the distance moved nor the heading is measured, the proposed solution is still usable. In this case, the maximum speed model is used to calculate a maximum walking range within the epoch time interval (the scenario “Assumed maximum speed” in Table 1). The subset of state candidates *C*_range_ defined by Equation (10) has higher transitional probabilities, while the others have lower transitional probabilities:

(10) $${\text{C}}_{\text{Range}}=\{j|\left\|P_{j}-P_{i}\right\|<\rho ,j\in 1,\cdots ,N\}$$

In this case, the subset of state candidates *C*_range_ is located within the whole circle area as shown in Figure 6(d). The radius of the circle is given by the range *ρ*.

In summary, this subsection indicates that the availability and accuracy of the measured MDI data have a direct impact on the calculation of transitional probabilities in the proposed solution. A robust positioning system should be able to recover a correct positioning result even if incorrect MDI data have been provided or erroneous positioning results have been produced in previous epochs. The proposed solution uses two practices to achieve robustness: First, it constraints all transitional probabilities to values greater than zero. In other words, Equation (3) is slightly modified as:

(11) $$a_{\mathrm{ij}}>0$$

Secondly, the scale *K* in Equation (5) is evaluated continuously with a changing value based on the reliability of MDI and the positioning solution of the previous epoch.

In contrast to previous studies that impose deterministic forms of motion models, the proposed HIPE solution measures the changing MDI using the smartphone sensors. Additionally, flexibility is important for the HIPE solution to work with different MDI combinations, thus it is usable with different smartphone platforms, which may have different types of sensors available for MDI measurements.

## 5. Experimental Evaluation

**Figure 7.**
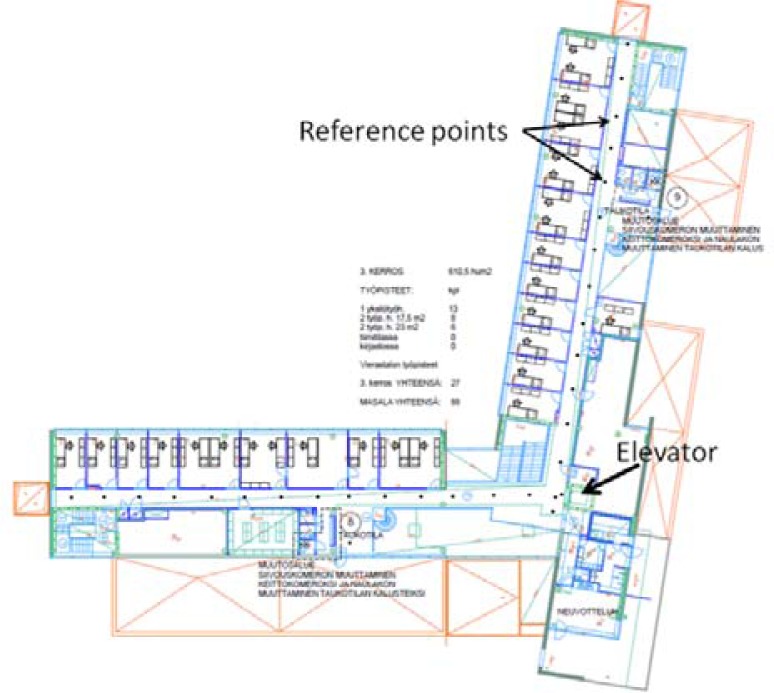
The layout and indoor environment of the experimental area.

The proposed HIPE solution was evaluated through a field experiment conducted on the third floor of an office building, occupied by the Finnish Geodetic Institute (FGI). The office building has a total of three floors, and it is a typical office environment, including corridors, office rooms, an elevator, staircases, and electronic devices, such as computers and printers. Figure 7 shows the layout of the building. The lengths of the two corridors are approximately 40 m each.

This section first evaluates the accuracy of the measured MDI data, *i.e.*, the distance moved and the heading, in an actual indoor environment. The experimental results provide the readers with perspective on the reliability of the smartphone sensors, and the accuracy values are then used as thresholds in the HMM positioning approach.

To evaluate the sensor measurements and the positioning results, three statistical error types are used, *i.e.*, the root mean square error (RMSE), the error mean (EM) and the maximum error (ME). These statistics indicate consistency between a measurement and its true value from different aspects.

For the compass and moved distance measurements, the error *ε* is calculated as:

(12) $${ɛ}_{t}=H_{t}-{\bar{H}}_{t}$$

where *H_t_* and *H̄_t_* are the measurement and its reference value at epoch t, respectively.

For positioning results, the error *ε* is calculated as:

(13) $${ɛ}_{t}=\left\|z(t)-\bar{z}(t)\right\|$$

where *z*(*t*) and *z̄*(*t*) is the positioning result and the corresponding reference at epoch *t*, respectively.

The calculations for RMSE, EM, and ME are defined as follows:

(14) $$\mathrm{RMSE}=\sqrt{\frac{1}{N}\sum_{t=1}^{T}{ɛ}_{t}^{2}}$$

(15) $$\mathrm{EM}=\frac{1}{N}\sum_{t=1}^{T}{ɛ}_{t}$$

(16) $$\mathrm{ME}=\mathrm{Max}({ɛ}_{t})\;\;\;\;\;\;t=1,2,\cdots ,T$$

where *T* is the number of epochs.

For the distance moved, a relative error rate is calculated for each test case to evaluate the case-by-case accuracy of the step length model:

(17) $$\xi_{l}=\frac{{ɛ}_{t}}{H_{t}}\;\;\;\;\;t=1,2,\cdots ,L$$

where *L* is the number of test cases.

A mean error rate is calculated for all cases to evaluate whether there is any bias in the step length model:

(18) $$\bar{\xi }=\frac{1}{L}\sum_{l=1}^{L}\xi_{l}\;\;\;\;l=1,2,\cdots ,L$$

### 5.1. Heading Accuracy of the Smartphone Compass

The smartphone compass was evaluated in a real indoor navigation scenario, where a tester held the device in hand and moved naturally in a manner of his or her choosing, which mean the tester can walk freely around the testing area and he/she can start or stop walking at any time. Before the experiments, the compass was calibrated by rotating the device in a number eight pattern for roughly one minute until it had the highest calibration level [47].

The true-north directions of the walking routes are adjusted with magnetic declination to obtain magnetic-north directions. The magnetic declination is the angle between magnetic north and true north, and its value can be acquired from [54] for a given geographical location and date. For the experimental area (Helsinki, Finland, August 2012), the current magnetic declination is 7°43′.

The obtained magnetic-north directions were used as the references for comparison with the compass measurements. The differences were considered as measurement errors, which can be caused by multiple factors, e.g., sensor noise, environmental disturbance, body swings of the tester, *etc.* This study is not intended to identify these factors or to reduce the errors. Instead, the experiment evaluated the uncertainty level of the smartphone compass in a real office environment.

Two testers each operated an experiment that included two motion states, walking and stationary. Table 2 shows the error statistics in terms of the RMSE, EM and ME of both tests. About 4,000 measurements were collected in each test. The results show that the measurements of the smartphone compass have a RMSE of approximately 10° during the stationary state and 30° during the walking state. This observation is consistent with those reported in previous studies [38].

**Figure 8.**
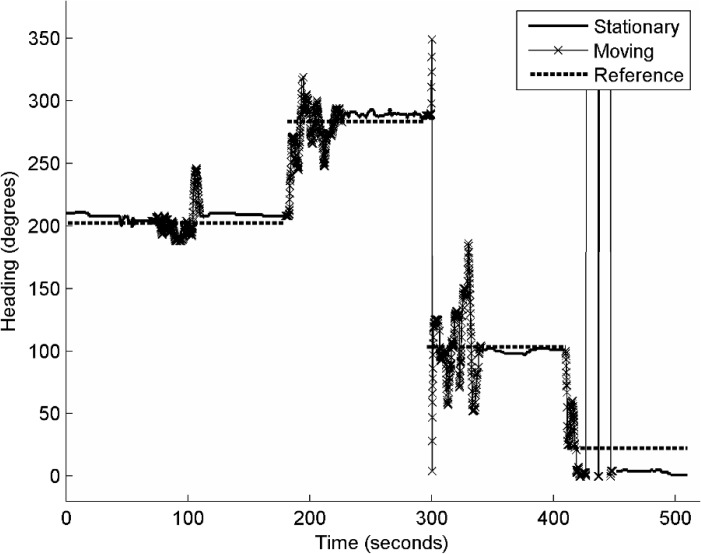
The heading measurements of the smartphone compass and the corresponding reference in an indoor environment.

Figure 8 further shows the epoch-by-epoch measurements of tester 1 and the reference used to investigate the error distribution over time. It can be concluded that the measurements are relatively smooth when the tester is stationary and significantly large errors and variations arise when the tester comes close to the elevator.

### 5.2. Accuracy of Pedestrian Distance Estimation

This experiment was conducted in a real pedestrian navigation scenario, and a pedestrian held the device in hand and moved naturally in a manner of his or her choosing. Within the same experimental environment, two testers each performed a test.

With measurements of the smartphone accelerometers, HIPE recognizes the current motion states, either stationary or walking. When it is recognized that the pedestrian is walking, HIPE counts the number of steps and further calculates the walking distance by multiplying the step number with an empirical step length of 0.7 m per step [43,44].

**Figure 9.**
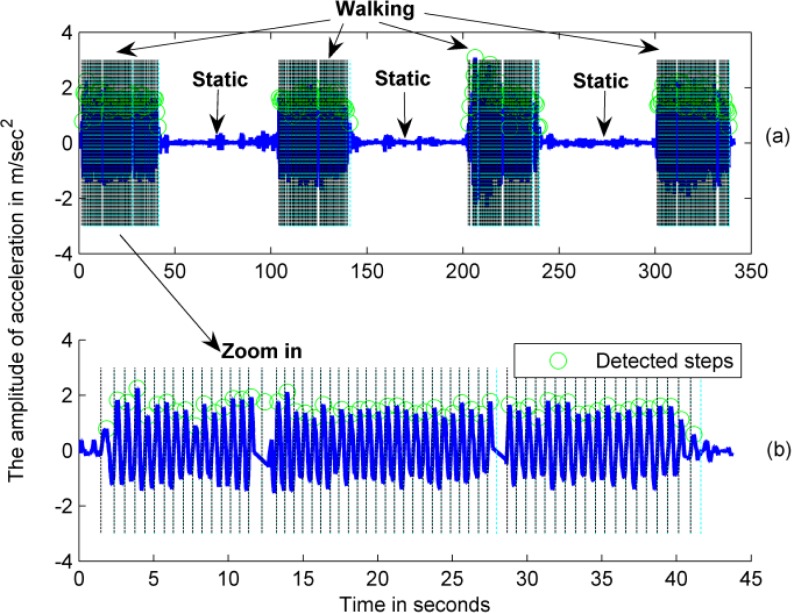
The results of motion state recognition and step detection based on the periodic acceleration pattern of a pedestrian. The blue line is the smoothed pedestrian acceleration, and the green circles indicate the detected steps.

Figure 9 shows the results of the motion state recognition and step detection for the first tester (Test 1). The test consists of three segments of a static state and four segments of walking. Figure 9(a) illustrates the whole process with the recognized motion states, and Figure 9(b) displays a magnified account of the first walking segment to show the detected steps in detail.

Raw accelerometer measurements are output at a rate of 35 Hz, and the blue line in Figure 9 indicates the smoothed pedestrian acceleration that was used to detect steps. The detected steps are shown as green circles in Figure 9.

Table 3 shows the errors of the derived walking distances of the two testers. Though illustrated only for two testers, the results clearly indicate that different pedestrians may have significantly different step lengths. Although the generic step length model used is appropriate for fitting the step length of different pedestrians and has a relatively small mean error rate of 1.86%, this model may have an error of approximately ±8% for the estimated walking distance of a specific pedestrian. The distance error is caused by step misdetection and the difference between the individual’s actual step lengths and the generic step length model.

It can be observed that recognition of the static state is highly reliable because there is no walking state detected during static segments. This means that, when HIPE recognizes the current motion state as static, the result can be used with high confidence. For example, the ratio *K* in (5) can be given with a greater value when the current state is recognized as static, as shown in Table 4.

To enhance the robustness of the positioning solution, the HIPE solution can tolerate a larger error in the distance estimate than the above experimental results. As shown in Table 4, HIPE assumes a relative error range of ±10% for a distance estimate and a least absolute error of 1.5 m that is half the separation distance of most RPs in the experimental area considered.

### 5.3. Positioning Results

Using the accuracy results of the MDI data evaluated in Sections 5.1 and 5.2, the proposed smartphone positioning solution was tested in the office environment described above. The tester held the smartphone and moved around in the test area in a manner of his choosing so that he could start and stop walking at anywhere any time. The experiment spanned for more than 1,500 s with approximately 160 RSSI observation epochs. This positioning test lasted for a longer period than the previous tests in Sections 5.1 and 5.2, and it did not use a predefined testing route for real performance evaluation. Figure 7 shows the experimental environment. Table 4 gives the values used for the parameters in the HMM solution.

For the error calculations, the time instance was recorded when each reference point was passed. The actual position of each RSSI observation epoch was then computed through interpolation utilizing the pedestrian dynamics information. The actual positions were used as the references in Equation (13) to calculate the positioning errors by epoch.

#### 5.3.1. Comparison of the Dynamic Positioning Results Using Different Combinations of MDI

As stated earlier, HIPE can work flexibly with different smartphone platforms, which may have different types of sensors available for the calculation of motion dynamics information. This subsection compares the positioning results that were produced by the grid-based filter algorithm using different combinations of MDI and the same RSSI measurements. The scenarios of different combinations of MDI are defined in Table 1. A common speed of 1 m/s was used to calculate a maximum walking range in the scenarios “Measured heading & assumed speed” and “Assumed maximum speed.”

**Figure 10.**
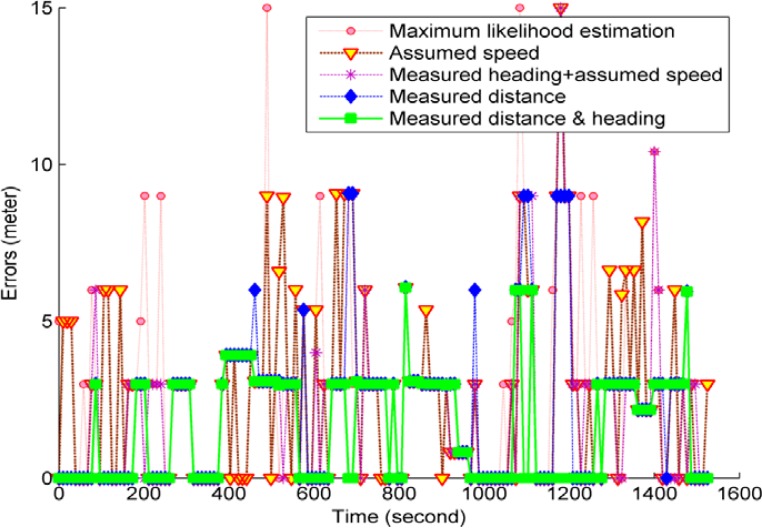
The epoch-by-epoch positioning errors of the HMM solutions and the MLE solution. The HMM solutions use different combinations of MDI as defined in Table 1 and utilize the grid-based filter algorithm to produce position estimates.

In this study, the proposed HMM solutions were compared with the classic method of MLE (maximum likelihood estimation). The MLE method is a classic fingerprint algorithm used in many previous studies [21,45,53,55], and it resolves location estimates with maximum likelihoods. Figure 10 illustrates the epoch-by-epoch positioning errors of the MLE method and the HMM solutions using different combinations of MDI data.

As shown in Table 5, the HMM solution in all cases has better performance than the MLE fingerprint algorithm. Figure 10 and Table 5 indicate that the MDI data improve positioning accuracy, and the positioning accuracy improves with increased use of MDI data. When different combinations of the MDI data given in Table 1 were used, the grid-based filter achieved an RMSE improvement of 1.34 m (30.3%), 1.26 m (28.4%), 0.95 m (21.4%), and 0.56 m (12.6%), and an EM improvement of 1 m (32.6%), 0.93 m (30.3%), 0.77 m (25.1%), and 0.51 m (16.6%) over the MLE, respectively.

**Figure 11.**
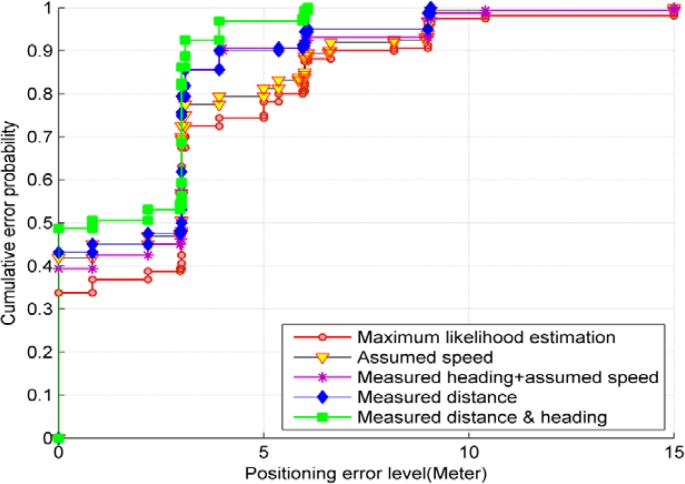
The cumulative probability distributions of the positioning errors related to the HMM solutions and the MLE solution. The HMM solutions use different combinations of MDI, as defined in Table 1, and utilize the grid-based filter algorithm to produce the position estimates.

To gain insight into the distribution of the positioning errors, Figure 11 presents the empirical cumulative probabilities of the positioning errors of the different cases. The comparison further shows that the MDI data effectively reduce the positioning errors. When more MDI is used, large positioning errors can be mitigated significantly and positioning reliability is improved.

#### 5.3.2. Positioning Accuracy of the Viterbi Algorithm *vs.* the Grid-Based Filter

As previously described, both solutions of the HMM problems, *i.e.*, the Viterbi algorithm and the grid-based filter, can be used to estimate positions, and each is suitable for different location-based applications. HIPE implemented both of these algorithms.

**Figure 12.**
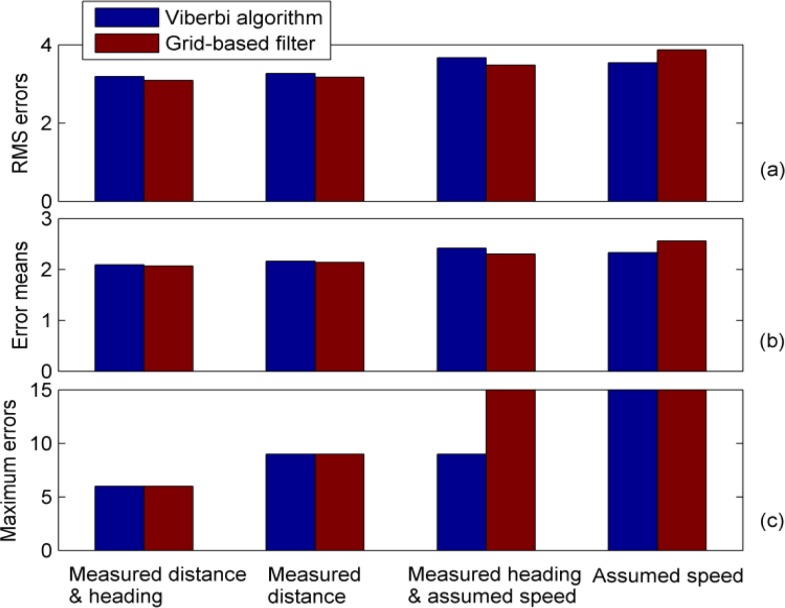
A comparison of the positioning accuracy of the Viterbi algorithm and the grid-based filter in terms of (**a**) the RMS errors, (**b**) the error mean and (**c**) the maximum errors. Both algorithms use different combinations of MDI, as defined in Table 1, and as specified at the bottom of this figure.

Figure 12 compares the positioning accuracy of the two algorithms, which use the same RSSI measurements and different combinations of MDI, as defined in Table 1. This figure shows that the statistics of the positioning errors have only slight differences in all cases. It is found that the Viterbi algorithm and the grid-based filter have comparable positioning performances when the same set of MDI data is applied.

## 6. Conclusions

This paper presented a smartphone indoor positioning engine named HIPE. Because the operation of HIPE only uses the built-in hardware and computational resources of a smartphone, the positioning solution presented here is more cost-efficient and convenient for integration with related applications and services than alternative systems presented previously.

The proposed HIPE solution is a hybrid solution, fusing multiple smartphone sensors with WLAN signals. The smartphone sensors are used to measure the motion dynamics information of the mobile user, and the MDI data augment the WLAN positioning by mitigating the impact of RSSI variance. In this paper, two algorithms of HMM problems, *i.e.*, the grid-based filter and the Viterbi algorithm, were used for data fusion to resolve the position estimates. Both algorithms demonstrated comparable positioning accuracy and are suitable for different types of applications.

In comparison to previous studies, which have commonly used deterministic motion models, the proposed HIPE solution is more widely applicable for various motion scenarios because it measures actual motion dynamics using smartphone sensors. The experimental results of the indoor positioning experiment showed that HIPE has adequate positioning accuracy and reliability. The accuracy of the positioning solution increased with increasing usage of MDI data.

The HIPE was implemented in this paper with the Nokia N8 smartphone, and it can be transferred to different smartphone platforms, even if such platforms utilize different combinations of sensors for MDI data measurement. This paper has presented the methods used to address different scenarios in which the various types of MDI are available.

In the future, other smartphone sensors, such as cameras and gyroscopes, will be integrated with HIPE to measure MDI. Three novel LBS smartphone applications, such as iParking [56], will be developed based on HIPE for demonstrations related to route guidance and indoor navigation in city ecosystems.

## Figures and Tables

**Table 1.** Different scenarios using various combinations of MDI.

**Combinations of MDI**	**Sensors and methods used to obtain MDI**	
	**Distance**	**Heading**
Measured distance & heading	accelerometers	compass
	accumulated step lengths	directly measured
Measured distance	accelerometers	---
	accumulated step lengths	unknown
Measured heading & assumed maximum speed	---	compass
	a constant speed model of 1 m/s	directly measured
Assumed maximum speed	---	---
	a constant speed model of 1 m/s	unknown

**Table 2.** The smartphone digital compass error statistics for stationary and walking states.

		**Test 1**	**Test 2**
**Stationary**	RMSE (°)	9.50	12.24
	EM (°)	−0.33	−6.02
	ME (°)	21.18	35.82
	Number of measurements	2984	2686
**Walking**	RMSE (°)	27.25	26.59
	EM (°)	−5.72	−5.06
	ME (°)	174.30	165.71
	Number of measurements	1420	1265

**Table 3.** The evaluation results for step detection and the distance moved estimation in the indoor office environment.

		**Tester 1**				**Tester 2**			
		1	2	3	4	5	6	7	8
Duration (s)		40.2	39.7	38.8	38.9	35.7	41	35.4	35.9
Step number	True	61	62	60	60	55	57	57	56
	Estimated	60	59	60	58	54	55	53	55
True distance		39	39	39	39	39	39	39	39
Estimated distance		42	41.3	42	40.6	37.8	38.5	37.1	38.5
Error of the estimated distance	Error rate	7.7%	5.9%	7.7%	4.1%	−3.1%	−1.3%	−4.9%	−1.3%
	Mean error rate	1.86%							

**Table 4.** The parameter settings used in the HMM solutions.

Tolerated motion distance error	±10% of a distance estimate, at least 1.5 m
Tolerated heading error	55°
*K* (the ratio between high and low transitional probabilities)	[200, 200,000]

**Table 5.** The positioning error statistics of the grid-based filter algorithm using different combinations of MDI (unit: m).

**Applied motion dynamics**	**RMS error**	**Error mean**	**Maximum error**
Measured distance & heading	3.09	2.07	6
Measured distance	3.17	2.14	9
Measured heading & assumed speed	3.48	2.30	15
Assumed speed	3.87	2.56	15
MLE	4.43	3.07	15
